# Building antimicrobial stewardship capacity through participatory health literacy workshops in Zimbabwe

**DOI:** 10.1093/jacamr/dlaf255

**Published:** 2026-01-23

**Authors:** Martin Mickelsson, Tungamirirai Simbini

**Affiliations:** Department of Earth Sciences, University of Gothenburg, Box 460, Gothenburg 40530, Sweden; Department of Women’s and Children’s Health, Uppsala University, Uppsala University Hospital, Uppsala SE-751 85, Sweden; Department of Biomedical Informatics and Biomedical Engineering, University of Zimbabwe, P.O. Box A178 Avondale, Harare, Zimbabwe

## Abstract

**Background and objectives:**

Antimicrobial resistance (AMR) poses a mounting sustainability challenge to healthcare systems, especially in Southern African settings, where antimicrobial stewardship capacity is limited by resource constraints, with structural challenges exacerbating the problem of resistance. Strengthening education could support the development of AMR-related knowledge, and stewardship skills for health practitioners are key to enhancing antimicrobial use and addressing AMR. This paper investigates how participatory research workshops can support the development of AMR-related health literacy among Zimbabwean health practitioners (doctors, nurses and pharmacists) and how such literacy can promote antimicrobial stewardship.

**Methods:**

Eight interdisciplinary workshops involving 25 health practitioners were conducted at two teaching hospitals in Harare, Zimbabwe. Workshop transcripts were analysed using a combination of a value-creation framework and health literacy. The analysis identified how workshops created immediate, applied and transformative values, supporting stewardship.

**Results:**

The workshops created, based on self-reporting from participants, values enabling practitioners’ development of AMR-related health literacy. Functional literacy could strengthen prescribing practices and patient adherence to treatment. Interactive literacy may improve interdisciplinary collaboration. Critical literacy have the potential to support the identification of drivers of AMR in resource-limited contexts in Southern Africa.

**Conclusions:**

Created values and AMR-related health literacy may support antimicrobial stewardship, with workshops providing a context-relevant approach to enhance AMS capacity in Southern African healthcare settings. This educational approach has the potential to contribute to bridging the gap between awareness and stewardship practice. Through integration into professional training, it could support the promotion of sustainable antimicrobial use in Southern African contexts.

## Introduction

Antimicrobial resistance (AMR) is a global health challenge disproportionately affecting Southern African countries where resource constraints, unregulated antibiotic use and fragmented health systems exacerbate the challenge.^1–4^ Zimbabwe, similar to many Southern African countries, face widespread resistance, yet there is limited empirical research studying the contextual conditions that shape efforts to address AMR.^5–7^ Educational efforts on AMR often narrows its focus on individual behaviour and seldom accounting for the systemic and social determinants impacting resistance, including poverty, economic pressures and weak health governance systems.^(8–11)^ Developing antimicrobial stewardship requires educational approaches that engages with the drivers of resistance and promotes interdisciplinary collaboration between medical doctors, nurses and pharmacists.^12,13^ While the creation of shared understandings and collaborative practices is recognized as key to strengthen stewardship efforts to address AMR, participatory educational approaches to AMR remain mostly underexplored.^13,14^ This paper investigates the potential of participatory research workshops to strengthen AMR-related health literacy among interdisciplinary health practitioners, framing AMR as a contextual sustainable health challenge that requires reflective collaboration as well as shared understandings across groups of health practitioners.

We conducted workshops at two major teaching hospitals in Zimbabwe, a setting characterized by extensive resistance, lack of accessible diagnostics capacity and limitations in infection prevention as well as water, sanitation and hygiene (WASH) infrastructure.^3–5,15^ These conditions impact everyday antimicrobial stewardship and decision making, highlighting the need to develop contextually sensitive and shared understandings and practices around AMR. Analysis of data from the workshops was conducted using a combination of the value-creation framework and health literacy.^16–20^ The analysis explored how participating health practitioners made sense of AMR in the context of their everyday practices and the contextual conditions of Zimbabwean healthcare they work within. To this end, we sought to understand what values participatory research workshops could create for health practitioners in terms of AMR-related knowledge, skills and collaborative practices. Situating the study in Zimbabwe's healthcare system provides insights into how context-sensitive, interdisciplinary education can contribute to health practitioners AMR strategies, addressing gaps in both research and ongoing health practices, while acknowledging and engaging with systemic and structural conditions shaping antimicrobial use and stewardship in Zimbabwean healthcare.^2,3^

This paper investigates how participatory research workshops can support the development of AMR-related health literacy among Zimbabwean health practitioners (medical doctors, nurses and pharmacists) and how such literacy can promote antimicrobial stewardship. Two research questions guide the study:

- What values were created for health practitioners in the workshops?
- How did these values contribute to health practitioners’ AMR-related health literacy?

Shifting the focus towards shared meaning making and knowledge co-creation, the paper contributes to AMR education as a collaborative process beyond the limits of individual behaviour change.

## Methods

The paper operationalizes a participatory research methodology through two participatory research workshop series with Zimbabwean health practitioners, a method previously used in Southern Africa, supporting the method’s contextual relevance.^21–25^ Our participatory research approach aligns stewardship initiatives in African hospital contexts.^26^ Furthermore, the workshops drew on case vignettes as key workshop tool.^27–32^ Workshops were co-facilitated by the authors and held at Parirenyatwa University Hospital and Harare (Sally Mugabe) Hospital, both in Harare. A total of 25 health practitioners were purposively sampled with 10 medical doctors (four women, six men), two nurses (two women) and one pharmacist (one woman) at Parirenyatwa Hospital; eight medical doctors (three women, five men), two nurses (two women) and two pharmacists (two women) at Harare hospital.^33,34^ The sampling was made to ensure representation of key expertise and disciplinary backgrounds from paediatrics, obstetrics, medicine and surgery, supporting a space for knowledge co-creation between medicine, nursing and pharmacy.^34–36^ With the study focusing on exploring AMR-related health literacy as part of AMR education, the sampling of participants focused on health practitioners engaging directly with patients. As such, participant numbers achieved analytical saturation while reflecting staff availability and approvals from clinical directors at the two hospitals. While crucial for diagnostic AMR stewardship and surveillance, laboratory personnel are rarely involved in direct patient interactions, including decisions regarding prescriptions and providing guidance about antimicrobial use. Not including laboratory personnel was thus an intentional decision as they fell outside the scope of this study. However, we acknowledge that this limits the papers’ contribution as their inclusion could have offered insights into pathways for diagnostic stewardship as an important component of antimicrobial decision making. Workshop materials, including structure, case vignettes and discussion questions were piloted involving three Zimbabwean researchers with experience in health and sustainability research who reviewed workshop structure, case vignettes and discussion questions as well as linguistic clarity. Their feedback was used to refine the case vignettes for increased contextual relevance and re-align the discussion prompts with the aim of the workshops. Their comments and feedback informed the final workshops. Workshops were audio recorded and transcribed by the authors with support from contemporary notes for improved depth and accuracy in the transcripts. Analysis of the transcripts was guided by the analytical framework and analysis process outlined next that draws on both deductive and inductive components.^37,38^

### Workshop design

The data in this paper draw on the two initial workshops of the series, with workshops focusing on approaches and conditions for AMR education in professional health practices. Each workshop followed a shared structure, with an introduction and framing presentation, followed by three reflective sessions with guiding questions and a concluding plenary session. Guiding questions focused on the approaches to, and conditions for, AMR education, including AMR risks, challenges of antimicrobial stewardship and patient communication and contextual pressures around prescriptions. In addition, case vignettes highlighted recurring AMR challenges in Zimbabwe. The introduction and framing presentation focused on orienting participants and positioning the workshop within the series as well as outlining key concepts and case vignettes to be used in the reflective sessions (60 min). The reflective sessions (60 min each) had participants organized into small groups mixing senior and junior medical doctors and nurses as well as pharmacists, with each session structured along three steps, based on a reflective writing method, that emphasize learning in interactions between individual meaning making, sharing with peers and group discussions.^39,40^ First participants engaged in individual reflective writing based on guiding questions (10 min), which was followed by participants taking turns to share verbatim their written text (20 min) and finally a joint discussion where groups were tasked with discussing the questions and individual contributions with the aim of identifying common themes (30 min). The first reflective session focused on participants’ understanding of key AMR concepts, the second on their own patient-oriented health practices and the third engaged with case vignettes presented at the beginning of the workshop. During the plenary session (60 min) groups shared identified themes and provided mutual feedback, with the workshop concluding with joint reflections on the topic of the workshop. Workshops were co-facilitated by the authors who circulated among the groups to promote inclusive participation and highlight minority voices. Facilitation also included a degree of directing to ensure discussions was kept in line and relevant to the guiding questions. Through this facilitation approach and with the operationalization of reflective writing method, we were able to promote inclusive workshop discussions and mitigate professional hierarchies. This was further emphasized by using rotating reporting roles in the small groups to support balanced contributions, allowing multiple participants to report on group discussions and identified themes. Beyond the workshops, there was no systematic follow-up or observation of clinical practice. Results, including value creation and changes in behaviour, practice or institutional routines are thus based on participants self-reports as part of the workshop reflections and discussions.

### Analytical framework

Value-creation framework is operationalized to examine how participatory workshops generate value across time, as value trajectories, while health literacy are used to connect these values to how practitioners navigate structural barriers. Combined, these approaches enable analysis of knowledge co-creation and translation into practice.

### Value-creation framework

Drawing on Wenger, Traynor and De Laat, value creation is separated into several analytical categories, starting with the immediate value participants derive from the interactions of AMR educational practices.^16,17^ Value can furthermore be potential, representing knowledge capital that prepares the participants for possible future challenges, which are thus valuable and reassuring even if they are never put to the test. Third, there is applied value as the leveraging of contacts, networks, knowledge and expertise to affect changes in practice as part of specific situations. This value involves the adaptation and operationalization of immediate and potential value to new contexts. Fourth, by improving performance and achieving the valued being and doings of the participants, we might talk about realized value. Finally, through the learning process of evaluating AMR educational practices, participants might find value in transforming, expanding and reframing the purpose(s) of AMR education, what it means to achieve success in addressing AMR and thus redefine how it is measured and evaluated.

### Health literacy

Addressing AMR requires expanding health literacy beyond understanding and evaluating health information to incorporate social determinants of health.^41–44^ Recent health literacy research shifts the focus to structural barriers and social and economic factors such as poverty and limited healthcare access, aligning with preventive, equity-focused interventions.^18–20,45^ Health literacy influences patient-provider relationships, self-care and healthcare use.^46^ In AMR education, health literacy are operationalized in three categories, functional, interactive or critical health literacy.^18^ Functional health literacy involves the ability to understand and use health information to navigate healthcare systems. Interactive health literacy builds on functional literacy emphasizing communicative and interpersonal skills, enabling individuals to effectively engage in shared decision making with healthcare providers. Critical health literacy includes the capacity to identify and challenge social determinants of health and act to improve health outcomes. Integrated, these categories of health literacy create a comprehensive framework that encompasses individual skills and contextual conditions affecting health outcomes.^18–20,46^ This conceptual approach aligns with the paper’s methodology exploring value creation in participatory research workshops.^47^

### Analysis process

Transcripts were analysed by both authors using a six-step thematic approach, combining deductive and inductive components to identify, analyse and report themes within the data.^48–51^ The first step focused on reading of the transcripts to create overview and familiarity of participants’ discussions. This was followed, in the second step, by inductive coding where key sentences reflecting recurring topics in the discussions were identified. In the third step, transcripts were deductively coded using the analytical framework (value creation and health literacy) as guide. The two sets codes created by each author was in the fourth step compared and discussed for enhance clarity and to ensure analytical representativeness and grouped into themes representing forms of value creation and health literacy. As part of step five, identified themes were correlated with the transcripts to ensure that all key dimensions of the participants’ workshop discussions were accounted for as part of the themes. Illustrative examples were drawn from the material as part of the final, sixth, step to support our analytical argument. Through this iterative analytical process authors met regularly to refine codes, review interpretations and validate identified themes to support analytical rigour, transparency and to assure that results should correctly represent the participants’ workshop discussions.

### Ethical considerations

This research obtained ethical approval from the Medical Research Council of Zimbabwe (reference number redacted for review version, included in the cover letter). In planning the study, permission was sought to conduct workshops with health practitioners from the respective clinical directors at each hospital. The study adhered to the principles of informed consent and voluntary participation. Participants were provided with consent forms and information sheets detailing the research aims, the workshop process, as well as the collection and management of research data. Opportunities were provided for participants to raise any questions regarding the research and workshop before going through and signing the consent form, with further opportunities offered during the workshops. The global code of conduct for research in resource-poor settings was adhered to in the study.^52^ Participant codes were used, and no participant names were recorded as part of the data collection.

## Results

This section highlights results from the workshop discussions on how values were created for the participating health practitioners. Operationalizing the value-creation framework, the results show how the workshops were more effective in creating immediate and potential values, linking them to the creation of applied and transformational values. Meanwhile the creation of realized value in terms of improving practice performance creation as part of the workshops was more difficult to track as there was no systematic follow-up or clinical observation after the workshop meaning that any results indicating changes in behaviour, practice or institutional shifts were self-reported by participants and should be understood as perceived and intended. Results illustrate the potential and limitations of participatory research workshops, including processes of knowledge co-creation, in engaging with AMR challenges in practice.

### Immediate value (activities and interactions)

The workshops created immediate value through enhanced professional exchange among the health practitioners. Participants shared diverse insights, including personal experiences with antimicrobial misuse, poor adherence to treatment plans and the need for clear patient communication, as exemplified in the following quote:

In the consultation room, when you meet a patient, you just educate them on antimicrobial resistance. Most of the people actually don’t know. And coming from a doctor’s perspective, they actually listen when you tell them something like that. [Participant 2, Workshop 2 (Hospital 2)]

Participants brainstormed ways to engage community leaders and leverage social platforms to improve AMR awareness. Case discussions and open-ended dialogues engaged with rational antibiotic use, misuse of antimicrobials in farming, hand hygiene and prescription practices. As part of participants’ interdisciplinary discussions and collaborative problem solving, a focus on shared responsibilities and ownership for stewardship emerged as participants drew on their professional expertise and experience to analyse the cases, outline their roles in AMR education and stewardship as well as discuss areas of overlapping responsibility. Through these discussions, participants mapped how their practices could contribute to patient counselling, AMR education and stewardship, as illustrated in the following quote:

Every person that is within the chain is responsible. So, responsibility will only come after the education. […] So, even when you prescribe for your patients, because most likely, if I come to you, doc, and then I say, look, I have a flu, I need Amoxy, and you don’t give me Amoxy, I’m going to go to Dr Ryan so that he gives me one. Before we just say, no, I'm not going to prescribe Amoxy, we need to also explain why we’re not prescribing Amoxy. And I think that’s where if we conscientize also the consumer so that they will know what to ask for and not to ask for. [Participant 3, Workshop 2 (Hospital 2)]

Workshops thus created immediate value centred on problem solving, shared accountability, practical interventions and effective engagement with patients. Such collective reflection on antimicrobial prescribing and patient communication indicted the development of shared responsibilities and ownership for antimicrobial stewardship practices.

### Potential value (assets and knowledge)

The workshops created potential by expanding participants’ knowledge and perspectives on AMR and its underlying systemic, institutional and behavioural drivers. As seen in the following quote, participants’ reflections and discussions recognized the benefits of an interdisciplinary understanding of AMR:

Looking at the one health aspect I didn’t know most of the stuff that we talked about today, especially the animal parts, and the food processes, I only got to know that today I think it would be very valuable if we have more of these sessions with a multidisciplinary approach that would benefit us as a community. [Participant 1, Workshop 1 (Hospital 1)]

Participant discussed cultural practices, unregulated pharmaceutical sales and gaps in healthcare infrastructure. This illustrated their preparedness to engage with stewardship challenges within their institution. When discussing ‘One health’, this was the participants own framing of the intersections between human, animal and environmental health raised as part of the workshop discussions. Participants also shared narratives of AMR risks, including self-prescription, incomplete antibiotic courses and misinformation during disease outbreaks, underscoring the need for AMR education, illustrated by the following quote:

It’s quite convenient to unjustifiably prescribe these drugs, because, for example, like I said, our obstetrics and gynaecology department, we’ve got a huge number of patients. I cannot afford to keep patients for two, three weeks while I wait for them to get money for investigations. [Participant 5, Workshop 2 (Hospital 2)]

Beyond awareness, the workshops thus created knowledge of the practical challenges health practitioners encounter around antimicrobials and AMR.

### Applied value (changes in ongoing practice)

On the basis of participants’ reflections and self-reported initial efforts and practice changes, the workshops created a degree of applied value by integrating AMR awareness into the health practitioners’ everyday practices. Reflections included patient education on completing prescribed treatments and understanding the risks of antimicrobial misuse, as exemplified in the following quote:

In consultations, we could take time, maybe three minutes, to explain what AMR is, the appropriate use of the antimicrobial agents, and build rapport with our patients and trust that they understand that it’s not every disease that needs an antimicrobial agent for them to recover. [Participant 3, Workshop 1 (Hospital 1)]

Participants reported initial efforts and emerging institutional practice changes in working with patients around daily practices. These efforts indicated a step towards stewardship-oriented behaviour, aligning patient communication and prescribing practices. Beyond individual practice, changes were reported by participants of initial efforts in hospital routines. This included discussions and intentions regarding appointing AMR education coordinators, forming infection control teams to monitor antibiotic sensitivity and shifting to evidence-based prescribing supported by diagnostic testing, as highlighted in the following quote,

What we’re doing now is to do a blood culture test or an infection screen, blood culture chest X-ray during MCS rather than just prescribing blindly. Teaching the patients on methods of prevention, on how to not get the infection in the first place, teaching them on sanitation methods, just the basic hygiene, washing your hands after visiting the toilet and also washing your hands before taking a meal. [Participant 4, Workshop 2 (Hospital 2)]

As there was no follow-up after the workshops on participants’ self-reported institutional changes, these should be understood as participants’ perceptions or intentions.

In addition, participating health practitioners outlined intentions to adapt AMR education to different audiences. This reflects a shift away from prescriptive one-size-fits-all approaches towards dynamic AMR education, including strengthening context-relevance, acknowledging the experiences, values and priorities of patients, policymakers or community contexts. These reflections illustrate participants self-reported intentions to translate awareness into practice changes both individually and institutionally.

### Realized value (improved performance)

While long-term improvement was challenging to assess within the study timeframe, participants self-reported examples of realized value. These emerged as part of the workshop discussions regarding clinical practice and stakeholder engagements. Participants expressed perceived improvements in terms of clarity regarding the roles and responsibilities of various stakeholders in AMR prevention, including healthcare professionals, policymakers and community leaders. This is exemplified in the following quote:

Engaging the policymakers, we need to engage them into this AMR education. Because we [health practitioners] can’t do this alone, this is actually bigger than any of us. We need those that made those policies to also be sensitized to the fact that if we do not take heed we might in the future not have any medical drugs to be using actually. […] to implement AMR in the workshops, we need equipped institutions. [Participant 7, Workshop 2 (Hospital 1)]

Participating health practitioners also reported their views on how the ‘whole society’ approach to AMR education had the potential to improve stewardship practices when integrating public health efforts with education, and sanitation, as exemplified in the following quote:

As much as we are trying to educate the people on preventing AMR, but the people are still living in the poor houses with lack of sanitation, so the bottom line is, as much as we educate them, yes we are going to see change, but still we need to change whatever is causing the emergence of antimicrobial resistance, we need to prevent, so there is need to involve the government and to put policies in place such that people have good housing, proper sanitation, good infrastructure, and proper waste disposal. [Participant 6, Workshop 2 (Hospital 2)]

These discussions and self-reported insights also extended to engaging with healthcare policies and routines as part of the everyday operations of the hospitals in the study, indicating how realized value could contribute to stewardship improvement.

### Transformational value (redefining success and ‘good’ practices)

From the participants’ self-reported insights and efforts, the workshops created transformational value by shifting how participants conceptualized AMR and how to address the challenge in practice. AMR education was redefined in the participants perceptions as an ongoing, systemic process to a systemic process rather than isolated clinical efforts, aligning with broader public health and sustainability goals as exemplified here:

We will all be responsible for AMR education. It will target healthcare workers and the public in general. Education will happen in the lecture rooms, as well as areas of practice, that is the hospitals, clinics, our private surgeries, etc. Then AMR education will be a holistic approach, targeting patient habits and also health care workers’ habits. [Participant 2, Workshop 1 (Hospital 1)]

Practitioners’ perceptions broadened beyond individual prescribing to systemic engagements with resistance and stewardship. As seen in the following quote, a key transformational value reported by the participants was the shift from reactive treatment to proactive prevention to avoid the emergence of resistance.

The main issue is we are very focused on treatment, we’re very focused on when the problem is there then we try to deal with it and to try and change our mindsets to whether is there a way that we can stop this from happening in the first place is very difficult. [Participant 1, Workshop 1 (Hospital 2)]

As part of the shift to prevention of infections driving resistance, participants perceived AMR not just as a medical or biophysical issue but as a societal challenge, affected by societal determinants of health. Improved sanitation and water access and infrastructure, reduced hospital-acquired infections and strengthened community engagement were discussed as redefined metrics of success for AMR education, as seen in the following quote:

For us to achieve what we are aiming to achieve, there are other issues that need to be addressed. We are saying the health facility is supposed to be fully built for it to provide the services that are required for us to reach an ultimate goal of prescribing the actual medication for the, be it in the wound infections, be it in the complicated deliveries, be it in organ transplant. We need to address issues in water, sanitation, waste management, and health policies. [Participant 5, Workshop 2 (Hospital 1)]

Participants proposed how transformational value could be created by integrating AMR awareness and prevention into doctors’, nurses’ and pharmacists’ training, promoting long-term adoption in healthcare. Through these reflections, participants redefined AMR education success from reactive treatment metrics to systemic prevention towards more sustainable, holistic approaches resistance management. Participants reported how workshops facilitated reflection on linking everyday clinical work with the structural and societal conditions of stewardship, generating, according to their reporting, a progression of value and illustrating an evolving engagement with AMR.

## Discussion

Through the workshops, health practitioners were able to critically engage with AMR by applying knowledge in ways that create value both individually and collectively: values that contributed to the health practitioners’ AMR-related functional, interactive and critical health literacy. These results align with previous research regarding the importance of health literacy for strengthening AMS, with functional literacy supporting prescription and patient counselling, interactive literacy facilitating interdisciplinary collaboration and stewardship networks and critical literacy enabling engagement with structural drivers of AMR.^6,11,13,18,19,46^ However, developing health literacy alone has been shown to not always be enough, especially in resource-constrained health care settings.^15,53^ By contrast, this study has sought to overcome these limitations by embedding literacy development as part collaborative and reflective participatory workshops that create spaces for health practitioners to reflect on prescription and patient communication and how these practices are affected by contextual conditions.

Extending beyond the prevalent focus on individual knowledge and behaviour in AMS, the paper illustrates the potential of participatory workshops to enable collaborative approaches to AMS and AMR education that accounts for contextual conditions of AMR.^53,54^ While previous participatory research on AMR has focused on communities and diverse societal stakeholders, this paper presents findings regarding value creation and health literacy development among health practitioners, suggesting how the participatory workshops detailed can potentially strengthen inter-professional collaboration within hospitals and enhance AMS efforts.^12,26^ This highlights the impact of methodological differences for participatory research outcomes and how focusing on health practitioners can result in specific forms of value creation. Crucially, these outcomes reflect participants’ reflections and self-reported changes in practices in which intersecting ecological, agricultural and infrastructural aspects of AMR and antimicrobial stewardships were highlighted. The relationship from created values through health literacy outcomes and potential interventions to potential stewardship contributions is outlined in Table 1. Together, the three forms of health literacy form the basis for how we understand health literacy that go beyond individual skills and encompass engagement with the social conditions and institutional circumstances of AMR as an area of education and a sustainability challenge.^18,19,46^

**Table 1.** Mapping the connections from workshop value creation through health literacy outcomes and potential interventions to potential stewardship contributions

Workshop values		Health literacy outcomes		Potential intervention		Potential Stewardship contributions
Immediate value: (case discussions, exchanges, and joint problem solving)	>	Functional literacy: improved ability to understand and communicate AMR concepts; simplified patient explanations.	>	Conduct structured case vignette-based discussions to co-create ways to engage and communicate with patients around AMR.	>	Could support more consistent patient counselling on antibiotic use; improved adherence support; clearer explanations of why antimicrobials are not always needed.
Potential value: (new knowledge, interdisciplinary perspectives, recognition of systemic drivers)	>	Interactive and functional literacy: strengthened inter-professional collaboration and shared understanding of AMR.	>	Facilitate interdisciplinary reflection sessions where health practitioners analyse systemic drivers of antimicrobial use and collaboratively outline priorities in promoting stewardship.	>	May enhance interdisciplinary collaboration; stewardship networks.
Applied value: (integrating AMR into consultations, adapting education for audiences)	>	Interactive and critical literacy: translation of knowledge into practice; adapting communication to local contexts.	>	Use reflective writing to develop contextually relevant ways to implement AMR communication and stewardship approaches,	>	Could contribute to reduced unnecessary prescribing; integration of AMR into hospital routines.
Realized value: (practice changes, reduced overprescription)	>	Critical literacy: ability to evaluate and challenge systemic barriers; acting collectively on AMR drivers.	>	Hold small group discussions for participants to share reflections on their roles in stewardship efforts and self-reported changes in clinical practice.	>	May support more evidence-based prescribing (e.g. use of diagnostics before prescribing); involvement in hospital- and policy-level AMS initiatives.
Transformational value: (reframing AMR as systemic, societal challenge; emphasis on prevention)	>	Critical literacy: reframing AMR as a public health and sustainability issue beyond the clinic.	>	Guide participants in plenary discussions where identified themes are shared with the goal of linking insights with clinical experiences and structural challenges, such as WASH, to enable ‘reframing’ of AMR as a systemic and preventive issue.	>	Could shift stewardship focus from reactive treatment to proactive prevention (WASH, sanitation, policy advocacy); ‘One health’ holistic stewardship strategies.

Functional health literacy focuses on gathering and developing foundational health knowledge and the ability to make key decisions were encompassed by the immediate value and potential value created as part of the workshops. These values contributed to functional health literacy in terms of skills development, where practitioners learned to simplify complex health concepts for patients, improving their ability to counsel patients on completing antibiotic courses and avoiding misuse. This aligns with how previous studies on antibiotic literacy and AMR education have highlighted the importance of such engagements with patients and especially contextually relevant communication approaches.^15,43^ These self-reported participant results indicate how functional literacy can support AMS capacity, including limiting demand for unnecessary antimicrobial prescriptions and improving patients’ adherence to treatment, thus encompassing practical solutions for resource-limited settings such as the Zimbabwean healthcare system. Meanwhile, these examples are participants’ reflections and perceptions of initial practice changes and potentials rather than long-term changes of clinical practice. While previous AMS studies focus on externally measures of behavioural change, our workshops encompassed reflective writing and discussions and facilitated inter-professional engagements that could explain the findings of how participants developed their reported intentions and initial efforts of practice and institutional change.^15,53–55^ An emergent theme was also how, seen from an interdisciplinary perspective and highlighted by the participants, functional literacy can also enhance communication between medical, veterinary and agricultural practitioners sharing responsibilities for across sectors.

The workshops fostered interactive health literacy through the creation of potential and applied value as part of collaborative knowledge co-creation and systems-thinking approaches. This included practitioners’ interdisciplinary engagements and community-oriented AMR education. Medical doctors, nurses and pharmacists developed shared knowledges and practices to address AMR, illustrating improved readiness, reported by the participants, to collaborate across professional boundaries. While previous research has highlighted the challenges of disciplinary hierarchies as obstacles to collaboration, including limited engagement by nurses, the results of the paper indicate the potential in structured process of reflective writing, sharing and facilitated discussions as part of participatory workshops to enable more inclusive participation knowledge co-creation.^13,36^ This indicates that methodological choices and facilitation techniques have the potential to mitigate hierarchical obstacles to AMS. These interactions created valuable professional networks committed to transforming AMR educational practices through collective action. Key to this process was the emphasis on contextual adaptation and community engagement while considering cultural and social factors influencing antimicrobial use. Although these factors have been part of previous research, our results indicate the potential of addressing them using methods of facilitated reflective writing and peer sharing as part of AMR education.^6,10,26,39,40^ This systems-thinking approach moved beyond prescriptive applications of AMR knowledge to responsive adaptations for real-world settings. By integrating these social aspects with environmental and agricultural realities, as discussed by participating health practitioners, stewardship becomes a shared societal task. In terms of building AMS capacity, interactive literacy re-framed stewardship as a collaborative rather than individual responsibility. Health practitioners self-reported how interdisciplinary collaborations and stewardship networks had the potential strengthen efforts in engaging with AMR as a complex socio-cultural issue requiring coordinated, context-relevant, approaches.

Applied and realized values contributed to critical health literacy enabling participants to analyse systemic barriers and translate knowledge into practice. Through discussions on antimicrobial misuse in agriculture, unregulated sales and infrastructure gaps, participants developed AMR strategies. They were able to engage in systemic advocacy, calling for pharmacy regulations and community education while adapting AMR education to consider social, economic and infrastructural conditions. Beyond acknowledging and describing structural drivers of AMR, the results highlight how participatory workshops can create opportunities for participants to connect everyday health practices with determinants of health.^10,56^ In contrast with previous research, this study did not include follow-up processes and long-term monitoring, which is reflected in how applied and realized values take the form of self-reported intentions and initial efforts on the part of the health practitioners. This highlights that while participatory workshop can develop literacy and catalyse intentions, sustained AMS practice change might require institutional engagement and integration.^6,7,11,12,55^ As part of a more structural perspective of stewardship, critical literacy enabled participants to engage with systemic drivers and identify how behaviour and practice change always occurs as part of broader contextual and institutional conditions.

This paper explored values created in participatory research workshops and their contributions to participating health practitioners’ AMR-related health literacy. Several limitations are acknowledged. First, the workshops were held over a 4-month period, focusing on participants’ experiences and reflections. Without post-workshop follow-up or clinical observations, insights regarding changes in behaviour or clinical practice in the form of applied and realized values are based on participants’ self-reported initial changes and initiatives. There is thus a limitation in evaluating sustained implementation and change. Second, the workshops were conducted at two teaching hospitals in Harare. This poses a limitation in that participants’ discussions on experiences and contextual challenges may not account for conditions in rural health care institutions where resource and staff limitations could influence prescribing practices, antimicrobial stewardship and AMR education. Third, participant sampling intentionally focused on health practitioners engaging with patients while excluding laboratory personnel. While laboratory staff have a key role in diagnostics and AMR surveillance they do not routinely engage in these patient-facing competencies and were thus outside the scope of the paper. Their exclusion limits analysis of diagnostic AMR stewardship and relations between laboratory and clinical practices. Future research could include laboratory staff in broader participatory research that specifically engages with diagnostic AMR stewardship. This could be achieved by conducting workshops including both clinical and laboratory health practitioners as well as observational studies focusing on the links between clinical prescription practices and laboratory diagnostic practices. Fourth, we sought to address interdisciplinary hierarchies though group composition and inclusive workshop facilitation. actively highlighting all participants contributions. However, power dynamics can still have affected the workshop discussions and the results. These limitations emphasize the need for future research that includes diagnostic laboratory personnel, extend the workshops over longer periods, incorporates clinical observation and evaluation to understand whether and how workshop-generated values translate into sustained and comprehensive antimicrobial stewardship practices across the health care system. Despite these limitations, the paper contributes to a growing body of research on participatory approaches to AMR and AMS by responding to calls for more equable AMR educational efforts and extending stewardship to account for social and structural drivers of resistance.^6,10,14,26,41,45^ Contrasting with previous AMS efforts, focusing either primarily on individual behaviour or processes at the system-level, the paper contributes with insights on how participatory workshops can assist in developing values and literacy among health practitioners, strengthen AMS in healthcare settings with limited resources and strengthen AMR education.

## Data Availability

The data supporting the results of this study are available from the corresponding author upon reasonable request.
